# Obstacles to Evidence-Based Procurement, Implementation, and Evaluation of Health and Welfare Technologies in Swedish Municipalities: Mixed Methods Study

**DOI:** 10.2196/45626

**Published:** 2023-06-15

**Authors:** Therese Norgren, Matt X Richardson, Sarah Wamala-Andersson

**Affiliations:** 1 Department of Health and Welfare Technology School of Health, Care and Social Welfare Mälardalen University Eskilstuna Sweden

**Keywords:** evaluation, evidence, health and welfare technology, implementation, mixed methods, municipalities, procurement, social care, effectiveness, Sweden, support, design, survey, interview, welfare, technology

## Abstract

**Background:**

Health and welfare technologies (HWTs) are interventions that aim at maintaining or promoting health, well-being, quality of life, and increasing efficiency in the service delivery system of welfare, social, and health care services, while improving the working conditions of the staff. Health and social care must be evidence-based according to national policy, but there are indications that evidence for HWT effectiveness is lacking in related Swedish municipal work processes.

**Objective:**

This study aimed to investigate whether the evidence is used when Swedish municipalities procure, implement, and evaluate HWT, and if so, the kinds of evidence and the manner of their use. The study also aimed to identify if municipalities currently receive adequate support in using evidence for HWT, and if not, what support is desired.

**Methods:**

An explanatory sequential mixed methods design was used with quantitative surveys and subsequent semistructured interviews with officials in 5 nationally designated “model” municipalities regarding HWT implementation and use.

**Results:**

In the past 12 months, 4 of 5 municipalities had required some form of evidence during procurement processes, but the frequency of this varied and often consisted of references from other municipalities instead of other objective sources. Formulating requirements or requests for evidence during procurement was viewed as difficult, and gathered evidence was often only assessed by procurement administration personnel. In total, 2 of 5 municipalities used an established process for the implementation of HWT, and 3 of 5 had a plan for structured follow-up, but the use and dissemination of evidence within these were varying and often weakly integrated. Standardized processes for follow-up and evaluation across municipalities did not exist, and those processes used by individual municipalities were described as inadequate and difficult to follow. Most municipalities desired support for using evidence when procuring, establishing evaluation frameworks for, and following up effectiveness of HWT, while all municipalities suggested tools or methods for this kind of support.

**Conclusions:**

Structured use of evidence in procurement, implementation, and evaluation of HWT is inconsistent among municipalities, and internal and external dissemination of evidence for effectiveness is rare. This may establish a legacy of ineffective HWT in municipal settings. The results suggest that existing national agency guidance is not sufficient to meet current needs. New, more effective types of support to increase the use of evidence in critical phases of municipal procurement and implementation of HWT are recommended.

## Introduction

Health and welfare technology (HWT) is described as technology-based interventions that aim at maintaining or promoting health, well-being, quality of life, and increasing efficiency in the service delivery system of welfare, social, and healthcare services, while improving working conditions of the staff [1]. Examples of HWT commonly used in Nordic countries include digital and GPS-based safety alarms, digital nocturnal supervision, remote physiological monitoring, and automated, home-based medication dispensers. Many of these technologies are not classified as medical devices, and therefore, do not fall under the jurisdiction of, for example, the European Union Medical Devices Regulation or United States Food and Drug Administration (USFDA) approval. Nonetheless, HWT has the potential to positively affect both independent and assisted health management and integrity of its users, particularly in elderly care settings [2,3]. Evidence for the effectiveness of HWT is often not well established, however, regarding both individual interventions [4,5] and related administrative processes [6,7].

Follow-up and evaluation of implemented interventions, both regarding the intervention itself and the implementation process, is highly prudent when faced with insufficient research-based evidence [8]. Municipalities in Sweden have nonetheless faced significant challenges when attempting to implement HWT, including a lack of structured implementation processes and uniform models for systematic evaluation and follow-up of municipal care [9]. To address some of these HWT-related challenges, the Swedish Association of Local and Regional Authorities (SALAR), a member organization for municipalities and regions, established a group of 10 “model” municipalities in 2020 regarding HWT implementation [10]. The group has received extra support from SALAR including external financing from the national government to assist in more effective implementation and use of HWT [11]. While health and social care services in these and other municipalities are mandated to use the best available knowledge and conduct evidence-based practice [12,13], HWT still appears to be widely implemented without applying these principles. Current national guidance on HWT use exists [7,14-17] but does not focus on evidence in such use.

This study’s objective was to answer the following questions: is evidence used in Swedish model municipalities’ procurement, implementation, and evaluation of HWT? If so, what kinds of evidence are used and how? Do these municipalities have the support they need to constructively use evidence when procuring, implementing, and evaluating HWT? If not, what support is desired?

## Methods

### Recruitment, Setting, and Participants

The setting for the study was Swedish municipal health and social care administrations. Of Sweden’s 260 municipalities, 10 of these were designated as model municipalities for HWT use by SALAR, and the study participants were recruited from this pool. The contact details of the appropriate civil servants in the model municipalities were provided by SALAR, and a request was sent by email to all regarding participation in the study. Officials of 5 municipalities agreed to participate in the study and, following a telephone-based information session regarding the study procedures, signed informed consent forms regarding their participation as representative municipal officials.

The population of the participating municipalities ranged from 57,000 to 125,000 inhabitants per municipality in March 2020. The municipalities were distributed geographically across the country. Overall, 2 of these were classified as “larger” and 3 as “smaller” according to national demographic classification levels [18].

### Study Design

An explanatory sequential mixed methods design was used in this study, with a quantitative and a qualitative phase [19].

#### Quantitative Phase

Quantitative data were collected first via a web-based survey in November and December 2021. The questionnaire was constructed using multiple-choice questions and individual open questions with free text (Multimedia Appendix 1) according to established guidelines [20]. Four aspects were addressed in terms of evidence use in respective sections of the survey: procurement, implementation, follow-up and evaluation, and desired support. The results of the multiple-choice responses and the open-question responses were then used to construct the basis for a semistructured interview guide in the subsequent qualitative phase.

#### Qualitative Phase

Semistructured interviews were then conducted with the same participants who responded to the initial web-based survey. The interview guides (Multimedia Appendix 2) contained common questions for all participants, but also municipality-specific questions dependent on each municipality’s responses in the survey.

### Data Analysis

According to the explanatory sequential mixed method, the quantitative and the qualitative data were analyzed separately and then amalgamated by interconnecting the data in the final analysis step [19]. For quantitative data, multiple-choice survey responses were compiled in a database, and analyzed and compiled descriptively in graphs and tables, for both individual and aggregated responses.

For qualitative data, content analysis was performed according to the method described by Graneheim and Lundman [21]. The primary researcher transcribed the interviews and structured these in a database. The units of meaning were then extracted, and the text condensed into target content corresponding to the study’s objectives. This text was encoded into code words, which were then categorized, with similar words and phrases collected into specific categories (Multimedia Appendix 3). No other statistical analysis of associations was performed due to the low number of participating municipalities.

### Ethical Considerations

The study was exempted from ethics approval requirements as participants were engaging in their roles as official municipal representatives responding to requests for information, and the methods were not classified by the national research ethics agency as human research attempting to affect the participants physically or psychologically. The study nonetheless used procedures that were in accordance with the Helsinki Declaration on research involving human participants. Participants provided informed consent (in Swedish) for the use of data under the Swedish right-to-information act, including that they were able to opt out of the study at any point. Participants’ contact information as municipal representatives was obtained from public sources in order to recruit and communicate during the conduct of the study. This information was not used in the analyses, publication, or its presentation however, and not stored after the completion of the study. No compensation was provided for participation in the study.

## Results

### Quantitative Phase

All municipalities (N=5) completed the quantitative phase in its entirety. All had implemented at least one HWT intervention in the past year, including digital alarm systems, digital medication reminders, and automated medication dispensers.

According to the survey responses, 4 municipalities had required evidence of effectiveness to some extent when procuring HWT interventions, although the frequency of such requirements across procurements was uncertain. The evidence was gathered mainly via referrals from other municipalities, CE marking, or via the supplier's own analyses. Two municipalities had an established model or process for the specific implementation of HWT interventions. Three municipalities had a plan for systematic follow-up and evaluation of HWT interventions. Three municipalities indicated that their municipal work unit desired support in how to procure, implement, and evaluate HWT interventions from an evidence perspective (Table 1).

**Table 1:** Summary of the responses regarding the survey’s main aspects (N=5).

	Requires evidence for effectiveness when procuring HWT^a^	Has an established process or model used specifically for implementing HWT	Has a plan for systematic follow-up and evaluation of HWT effectiveness	Follows up requirements established during procurement related to HWT effectiveness	Desires support for evidence use in HWT-related processes
Large municipality 1	Yes, rarely	Yes	Yes	Yes, rarely	Yes
Large municipality 2	Do not know	No	Yes	Yes, sometimes	No
Smaller municipality 1	Yes, often	Yes	Yes	Yes, often	No
Smaller municipality 2	Yes, sometimes	No	No	Yes, rarely	Yes
Smaller municipality 3	Yes, rarely	No	No	Yes, rarely	Yes

^a^HWT: health and welfare technology.

All studied municipalities responded that they conducted follow-up and evaluation of HWT interventions via staff directly using the technology. In total, 4 municipalities conducted follow-up and evaluation of interventions via other employee roles, and 2 municipalities via the supplier. Three municipalities reported that they used the results from follow-up and evaluation of HWT interventions to make necessary adjustments to improve the effectiveness of the technology, although this was done rarely, sometimes, and often in the respective municipalities. Two respondents did not know if the municipality was conducting follow-up and evaluation of interventions. Three municipalities disseminated results of their own evaluation of HWT intervention effectiveness, most commonly through internal reports and via the supplier.

### Qualitative Phase

All municipalities (N=5) completed the qualitative phase in its entirety. Ten categories emerged from the content analysis: (1) evidence via references from other municipalities when procuring HWT, (2) evidence via documentation from suppliers when procuring, (3) evidence via benefits realization when procuring, (4) difficulties obtaining evidence when procuring, (5) Plan-Do-Study-Act (PDSA) as a model used when implementing HWT, (6) organizational models when implementing, (7) value and benefits when evaluating implemented HWT, (8) survey use during follow-up and evaluation of HWT, (9) desire for practical tools and knowledge transfer regarding use of evidence in HWT-related processes, and (10) desire for support networks with other municipalities regarding evidence use and HWT.

The categories related to procurement (1 to 4) confirmed survey results that other municipalities were the most common source of evidence during procurement, and lacking a reliable source created difficulties. One civil servant described this accordingly:

The interviews revealed that those who worked directly with HWT were not involved in the procurement process to any meaningful extent, and they often did not have sufficient information about what was included in this process. One civil servant explained:

Municipalities expressed the need for evidence that was relevant to their own settings, and in particular, evidence regarding effect and benefit realization for the user, during the procurement process, but this kind of evidence was generally difficult to obtain.

The categories related to implementation (5 and 6) involved the use of implementation models in general, specifically the PDSA and organizational models. The PDSA model was considered more fit-for-purpose in enabling evidence use, although it was unclear if it was used for this purpose. The organizational model was described as having a clear structure with a steering group and external frameworks to be formed, but it lacked guidance for how projects should be implemented:

Other municipalities expressed the need for an established implementation plan that could be integrated into regular work processes:

The categories related to follow-up and evaluation (7 and 8) primarily addressed surveys and value or benefit evaluation. Evaluation via survey, including questionnaires to staff and users following implementation of HWT interventions, preferably in close collaboration with first-line managers, was considered potentially useful. The intention was that decision makers could use the data to follow working environment conditions and adjust working methods if deemed necessary. While national agency guidance on value and benefit realization was mentioned, respondents found it difficult to evaluate qualitative benefits, especially from the users' perspective, as well as long-term benefits. It was considered easier to evaluate economic benefits, time savings, and short-term benefits:

Municipalities in some cases had begun developing a framework for working with benefit evaluation and benefit calculations if one did not already exist. In one such municipality, adaptation of an externally developed evaluation model was being considered, in which they mentioned the potential for evidence production:

The categories regarding desired support (9 and 10) elucidated the desire for follow-up at different levels and in different ways, from the citizen or user perspective to personnel to the IT department. Three municipalities had indicated that this support was desired for their own organizations, while all municipalities had suggestions for support mechanisms that were not specific to their own organization. Support that was desired or suggested for using an evidence-based perspective is described in Table 2, related to processes and other categories.

**Table 2:** Desired or suggested support for using an evidence-based perspective.

Process steps and requested support		Type of support
**Preparation**		
	How to describe the current evidence level	Knowledge transfer
	How to set evidence-based goals	Knowledge transfer
**Procurement**		
	Templates for tendering documentation that uses evidence	Practical tools
	References regarding evidence-based interventions	Networking with other municipalities
	Legal aspects	Knowledge transfer
**Implementation**		
	Established plans	Practical tools
	Checklists for implementation	Practical tools
	Competence on how to lead implementation or change	Knowledge transfer
**Evaluation**		
	Competency in evidence-based follow-up	Knowledge transfer
	Competency in value or benefit realization in different groups and settings	Knowledge transfer
	Simple manuals for qualitative benefit evaluation	Practical tools
	Simplified tools for measurement	Practical tools
**Dissemination**		
	Create a common database for sharing evidence	Networking with other municipalities
	Create support networks for evidence use	Networking with other municipalities

## Discussion

### Principal Findings

Structured processes that consider evidence during procurement, implementation, and evaluation of HWT are partially or entirely lacking in Swedish municipalities that are leading the deployment of such technologies. These municipalities desire more support in using an evidence-based approach when procuring and implementing HWT.

Both quantitative and qualitative results showed that evidence was requested by 4 of 5 municipalities during procurement but only infrequently due to difficulties in formulating what and how it should be requested or required. In the cases where evidence was requested, references from other municipalities regarding evidence were considered most often, while supplier-provided evidence (when presented) was considered less trustworthy. Assessment of any provided evidence was most often conducted by personnel administratively responsible for the procurement process and in some cases the intended staff users of the procured technology. It was, however, reported that there was a gap between these staff groups. *The 3 municipalities that reported using a process or model for the HWT implementation did not clearly describe how evidence was used when describing it, and the remainder described the deficits in, or absence of, any implementation model (including non-HWT specific ones) in their organizations.* While follow-ups and evaluation processes existed in 3 municipalities, they varied in their methodology and were described as difficult and inadequate. Most municipalities desired support for their own organizations in using evidence across the addressed aspects of HWT. All municipalities suggested tools or methods for this kind of support across a broad spectrum of purposes and types, however.

These principal results demonstrate that although there is often interest in using an evidence-based perspective in municipal HWT–related processes, municipalities completely or partially lack structured processes and competency to use or generate evidence within these. This falls in line with previous assessments of knowledge about general scientifically based approaches in social service activities [22]. This may reflect the continued absence of national directives and recommendations regarding evidence-based HWT use in municipalities, but also the unawareness of or unwillingness to use existing national guidance on HWT that does not specifically address evidence [23].

The municipalities participating in this study were nationally designated “model” municipalities representing sector-leading HWT use, which would suggest that it is even less likely that other nonmodel municipalities use evidence-based approaches when using HWT interventions. The observed deficits in the use of a structured, evidence-based approach during key HWT-related processes run contrary to national mandates for municipal health and social care services for using the best available knowledge and conducting evidence-based practice [13,24]. It is furthermore conceivable that the lack of high-quality evidence from relevant settings may dissuade municipalities from continuing to request or require it during procurement, instead choosing to rely on other municipalities’ recommendations. However, the opportunity for municipalities to generate real-world evidence (RWE) when implementing HWT with uncertain *a priori* evidence in their own organizations appears to be largely missed due to variability in implementation methods. Logically, it seems difficult to ensure the use of evidence in the absence of such structured evidence-generating processes.

Two key research directions appear appropriate in amending such shortfalls. First, robust evidence from rigorous, controlled HWT intervention studies should increase. Second, the generation of RWE from deployed HWT interventions in health and social care settings should increase to demonstrate effects and be more readily disseminated. HWT-related research collaborations between municipalities, academia technology developers, care providers, and users should be encouraged to proceed in these 2 directions.

### Comparison With Prior Work

Previous research has highlighted the use of HWT despite the lack of evidence for effectiveness or safety [4-6], and the current results contribute to explaining why this may occur. Other research by Kuoppamäki [25], who found a lack of established strategies for using HWT in elderly care settings, as well as an emphasis on intermunicipal consultation when seeking knowledge and best practices for HWT, is also confirmed by the results in this study. The results also lend support to the perceived uncertainties in operational responsibility for implementation pointed out in an earlier report by the Swedish eHealth agency [23]. The recent annual report based on data from all 292 Swedish municipalities on the status of e-HWTs clearly reported a lack of systematic evaluation and lack of a specific plan for follow-up after HWT implementation [26].

Previous research also demonstrates that municipalities lack evaluation and specific plan for follow-up of HWT [17,26]. Additionally, these results support previous findings that illustrated a lack of consensus on how HWT should be evaluated [27].

### Limitations

Transferability and generalizability of results are largely limited to publicly funded settings that use HWT. The low number of participating municipalities may reduce the certainty in comparing the variation of responses with other municipalities. Current results related to public procurement of HWT may be less relevant for countries with health systems different from Sweden or other Nordic countries regarding financing, organization, and roles.

### Conclusions

The deficits in or lack of structured processes for considering evidence during procurement, implementation, and evaluation of HWT in leading Swedish municipalities may call into question the effectiveness of HWT interventions. It could also lead to persistent negative effects on working conditions, user health and well-being, and organizational efficiency. The cost-benefit of implementing many HWT interventions would thus be greatly reduced or produce deficits. Considering the lead times to implementation and the typically long life span of implemented technologies, the risk of creating a legacy of ineffective HWT interventions is considerable. Targeted research funding to conduct robust research based on experimental evidence and RWE for HWT interventions is justified. Generation of effective national guidelines and recommendations to support evidence-based procurement and implementation of HWT-related processes should be prioritized to provide better support to municipalities in their work.

## Appendix

Multimedia Appendix 1 Questions from the web-based survey (translated from Swedish).

Multimedia Appendix 2 Examples of questions in the semistructured interview guide (translated from Swedish).

Multimedia Appendix 3 Example of the qualitative content analysis method.

## Abbreviations

HWT: health and welfare technology
PDSA: Plan-Do-Study-Act
RWE: real-world evidence
SALAR: Swedish Association of Local and Regional Authorities
USFDA: United States Food and Drug Administration

## Data Availability

All data collected from the study are available, anonymized, and in structured format, upon request by email to the corresponding author.
