# Supplementary material for: Obstacles to Evidence-Based Procurement, Implementation, and Evaluation of Health and Welfare Technologies in Swedish Municipalities: Mixed Methods Study
Source: JMIR Form Res. 2023 Jun 15;7:e45626. doi: 10.2196/45626 (PMC10337388; doi:10.2196/45626)
Supplement: Multimedia Appendix 1 [file formative_v7i1e45626_app1.docx]

## Multimedia Appendix 1. Questions from the web-based survey (translated from Swedish).

## Section 1: Personal information (anonymized during analysis)

1.My name is:

2.My email address is:

3. My job title is:

4. My area of responsibility and/or duties within my organisation are:

## Section 2: Information about your organisation

5. The name of my organisation is:

6. My organisation’s work is mainly conducted within/in the following:

- A municipality
- Several municipalities
- A region
- Multiple regions
- Throughout the country
- Internationally

7. The target group of my organisation’s work is the following:

(multiple choices allowed)

- All residents
- Selected users and/or patients
- Decision makers and/or politicians
- Other organizational parts within the same organisation
- Other activities outside your organisation, e.g. authorities, executors
- Other (provide description)

8. My organisation’s main tasks include the following:

(multiple choices allowed)

- Primary care - municipal
- Primary care - regional
- Specialist care
- Social services/welfare services
- People's health
- Support for other organizational parts
- Administration
- Management
- Other (provide description)

## Section 3: Procurement of health and welfare technology (HWT)

*The following questions concern the procurement of HWT within your organisation. In your answers, assume a procurement of HWT that is advertised and where tenders are received and assessed. Also consider that it should be of a "normal" type for your organisation in terms of cost and the technology's requirement profile or function.*

*It is okay not to answer some questions if you are not familiar with the subject. It is also fine to forward the survey link to someone else in your organisation to answer them in their own response form.*

9. My organisation procures or is a customer of HWT.

- Yes
- No
- Do not know

10. My organisation procures the following types of HWT:

(multiple choices allowed)

- Journal or documentation system
- Triage or decision support systems
- Monitoring system for somatic measurements, e.g. heart rate, blood pressure, etc
- Supervisory or security systems, e.g. cameras or sensors for doors, beds, rooms, etc
- Aids for users/patients, e.g. medication reminders, cooking, hygiene, etc
- Remote care or remote communication system
- Alarm system e.g. security alarm, GPS alarm
- Health promotion applications for mobile devices
- Training or skills development system for staff
- Other (provide description)

11. My organisation requires evidence for the technology's effectiveness when procuring HWT.

*Remember: Evidence for effectiveness means evidence that supports that the technology leads to the desired or expected outcome(s).*

- Yes, often
- Yes sometimes
- Yes, rarely
- No never
- Do not know

12. During procurement, my organisation requires evidence for the technology's effectiveness regarding the following outcomes:

(multiple choices allowed)

- Improved or maintained somatic health
- Improved or maintained well-being
- Fewer unwanted events or deviations
- Increased cost efficiency
- More efficient workflows or processes
- Improved work environment
- Do not know
- Other (provide description)

13. In procurement, my organisation accepts the following materials as evidence for the effectiveness of the technology:

(multiple choices allowed)

- Published scientific studies
- Follow-ups or investigations carried out by consultants or third parties
- Follow-ups or investigations carried out by the supplier of HWT
- Proven CE marking
- Proven compliance with the EU Medical Device Regulation (MDR)
- Reference assignments from other buyers/orderers
- Do not know
- The organisation does not request such materials
- Other (provide description)

14. During procurement, my organisation assesses materials that constitutes evidence for the technology's effectiveness with the following resources:

(multiple choices allowed)

- One or more people with expertise in the field
- People within the organisation
- People from external organisations
- Responsible procurer / procurement group
- My organisation does not assess such materials
- Do not know
- Other (provide description)

15. In procurement, my organisation uses the assessment of materials that constitute evidence for the effectiveness of the technology for the following purposes:

(multiple choices allowed)

- Scoring when evaluating incoming tenders
- Decisions regarding qualification in the procurement process
- As a recommendation to the client or other party in the organisation
- Do not know
- Other (provide description)

## Section 4: Implementation of health and welfare technology (HWT)

*The following questions concern the implementation of HWT in your organisation. In your answers, assume implementation of HWT that has been procured or ordered via procurement by your organisation. Also consider that it should be of a "normal" type for your organisation in terms of cost and the technology's requirement profile or function.*

*It is okay not to answer some questions if you are not familiar with the subject. It is also fine to forward the survey link to someone else in your organisation to answer them in their own response form.*

16. During the past year, my organisation has implemented, or started implementing, HWT.

- Yes
- No
- Do not know

17. During the past year, my organisation has implemented, or started implementing, the following types of HWT:

(multiple choices allowed)

- Medical technology products
- Journal or documentation system
- Triage or decision support systems
- Monitoring system for somatic measurements, e.g. heart rate, blood pressure, etc
- Supervisory or security systems, e.g. cameras or sensors for doors, beds, rooms, etc
- Aids for users/patients, e.g. medication reminders, cooking, hygiene, etc
- Remote care or remote communication system
- Alarm system e.g. security alarm, GPS alarm
- Health promotion applications for mobile devices
- Training or skills development system for staff
- Other (provide description)

18. My organisation has an established process or model that is used specifically when implementing HWT.

- Yes
- No
- Do not know

19. My organisation has a plan for systematic follow-up and evaluation of the technology's effectiveness.

- Yes
- No
- Do not know

20. My organisation follows up on the requirements related to the technology's effectiveness that were specifically set during the procurement.

- Yes, often
- Yes sometimes
- Yes, rarely
- No never
- Do not know

21. My organisation uses the following approach to follow up the effectiveness of the technology during implementation:

(multiple choices allowed)

- Via the staff who work with the technology
- Via other employees within the organisation
- Via third or independent parties
- Via researcher / research effort
- Via the supplier
- My organisation does not follow up on the effectiveness of the technology
- Do not know
- Other (provide description)

22. My organisation disseminates the results from follow-up and evaluation of the technology's effectiveness in the following way:

(multiple choices allowed)

- Via scientific publication
- Via publication that is actively made available to the public
- Via external network or organization within the public sector
- Via report intended for internal use / feedback
- Via feedback or dialogue to third parties
- Via the supplier
- My organisation does not disseminate the follow-up of the technology's effectiveness
- Do not know
- Other (provide description)

23.My organisation uses the results of monitoring and evaluation of implemented technology to make the necessary adjustments to improve the effectiveness of the technology.

- Yes, often
- Yes sometimes
- Yes, rarely
- No never
- Do not know

24. In my organisation, I feel that evidence, follow-up and evaluation of the technology's effectiveness are prioritized.

- Yes
- No
- Do not know

## Section 5: Other questions

25.In your opinion, does your organisation need support in how to procure, systematically follow up and/or evaluate in order to be able to implement HWT with an evidence perspective?

- Yes
- No
- Do not know

26.If so, what kind of support would you like?

27. Feel free to provide more information or explanation about the questions you have answered in the survey, if you think it could be valuable.
