# Supplementary material for: Obstacles to Evidence-Based Procurement, Implementation, and Evaluation of Health and Welfare Technologies in Swedish Municipalities: Mixed Methods Study
Source: JMIR Form Res. 2023 Jun 15;7:e45626. doi: 10.2196/45626 (PMC10337388; doi:10.2196/45626)
Supplement: Multimedia Appendix 2 [file formative_v7i1e45626_app2.docx]

# Multimedia Appendix 2. Examples of questions in the semistructured interview guide (translated from Swedish).

## Section 3

Regarding the survey question: *My organisation requires evidence for the technology's effectiveness when procuring HWT.*

**QUESTION:** You answered that you "often demand evidence". Can you give an example of how this has been done and why?

- FOLLOW-UP QUESTION 2: How well, in your opinion, has it worked with the review or assessment of the evidence provided by the tenderers? Why?
- FOLLOW-UP QUESTION 3: How would you like to develop your work with such evidence, if at all?

*Alternative:*

**QUESTION:** You answered that you "sometimes demand evidence". Can you give an example of how it has been done and why?

- FOLLOW-UP QUESTION 2: How well, in your opinion, has it worked with the review or assessment of the evidence provided by the tenderers? Why?
- FOLLOW-UP QUESTION 3: Do you think it would be desirable to make such requirements more often in procurement? Why?
- FOLLOW-UP QUESTION 4 (if yes to follow-up question 3): What would be needed or facilitated for you to make demands for evidence more often?

*Alternative:*

**QUESTION:** You answered that you "rarely demand evidence". Can you give an example of how it has been done and why?

- FOLLOW-UP QUESTION 2: How well, in your opinion, has it worked with the review or assessment of the evidence provided by the tenderers? Why?
- FOLLOW-UP QUESTION 3: Do you think it would be desirable to make such requirements more often in procurement? Why?
- FOLLOW-UP QUESTION 4 (if yes to follow-up question 3): What would be needed or facilitated for you to make demands for evidence more often?

*Alternative:*

**QUESTION:** You answered that you "don't know if you require evidence". Who within your business would probably be able to ascertain whether evidence is used or not?

- FOLLOW-UP QUESTION 2: Do you think it would be desirable to make such requirements more often in procurement? Why?
- FOLLOW-UP QUESTION 3 (if yes to follow-up question 2): What would be needed or facilitated for you to make demands for evidence more often?

# Section 4

Regarding the survey question: *My organisation has a decided process or model that is used specifically when implementing HWT.*

**QUESTION:** You answered "yes". Can you describe the model, e.g. what are the main steps involved in the process and who is responsible for them?

- FOLLOW-UP QUESTION 2: How well, in your opinion, has the implementation model/process worked? Why?
- FOLLOW-UP QUESTION 3: How would you like to develop your implementation process/model, if at all?
- FOLLOW-UP QUESTION 4 (if yes to follow-up question 3): What kind of support would you need to develop such a model?

*Alternative:*

**QUESTION:** You answered "no". Do you consider that a process/model specifically for implementing HWT would be desirable? Why?

- FOLLOW-UP QUESTION 2 (if yes to the question): Are there any particular elements or steps that you have already thought you would like to include in the process?
- FOLLOW-UP QUESTION 3: What kind of support would you need to develop such a model?

Regarding the survey question: *My organisation has a plan for systematic follow-up and evaluation of the technology's effectiveness.*

**QUESTION:** You answered "yes". Can you describe the plan, e.g. what are the main steps included in the plan and who is responsible for them?

- FOLLOW-UP QUESTION 2: How well do you think the follow-up and evaluation plan has worked? Why?
- FOLLOW-UP QUESTION 3: How would you like to develop your follow-up and evaluation plan, if at all?
- FOLLOW-UP QUESTION 4 (if yes to follow-up question 3): What kind of support would you need to develop the plan?

*Alternative:*

**QUESTION:** You answered "no". Do you think that a plan for follow-up and evaluation of HVT would be desirable? Why?

- FOLLOW-UP QUESTION 2 (if yes to the question): Are there any particular elements or steps that you have already thought you would like to include in such a plan?
- FOLLOW-UP QUESTION 3: What kind of support would you need to develop such a plan?

# Final questions

- What support do you think municipalities need to facilitate the use of HWT?
- How do you see the municipality's future need to use HWT? What types of HWT do you think may become relevant in the coming years? Which intended target groups for these technologies?
