# Supplementary material for: Obstacles to Evidence-Based Procurement, Implementation, and Evaluation of Health and Welfare Technologies in Swedish Municipalities: Mixed Methods Study
Source: JMIR Form Res. 2023 Jun 15;7:e45626. doi: 10.2196/45626 (PMC10337388; doi:10.2196/45626)
Supplement: Multimedia Appendix 3 [file formative_v7i1e45626_app3.docx]

Multimedia Appendix 3. Example of the qualitative content analysis method.

| **Meaning-bearing unity** | **Condensed meaning-bearing unit** | **Code** | **Category** |
| --- | --- | --- | --- |
|  |  |  |  |
| But what I place the greatest value on in such a procurement is references, that you can contact other municipalities that already have the product or service. | Greatest value when referring from other municipalities | References | References from other municipalities |
| It is based on an improvement wheel, PGSA wheel. It consists of four phases or four parts. | Improvement wheel, PGSA wheel. | PGSA wheels | PGSA as a model |
| x number of weeks after commissioning, the first survey goes out to both staff and employees about how to experience the actual replacement of the system, the new technology. Everything from whether you have gained sufficient knowledge of how it works, that you have gained ..., really how smoothly everything has gone but also focused on the working methods and how you look at that part. | Questionnaire to staff about the experience of the exchange of the new technology, whether it has gone smoothly, sufficient knowledge, etc. | Survey staff | Follow-up via surveys |
| Simplified tools but at the same time are not too simple, because then there is a risk that you miss something, but something that you can both use at the organizational level, but which we can also give to our unit managers so that they can become more active in this work. | Simplified tools for organizational level and unit managers | Simplified tools | Practical tools |
